# Preoperative Cardiopulmonary Exercise Testing and 30-Day Postoperative Complications After Lung Resection for Non–Small Cell Lung Cancer: A Retrospective Cohort Study

**DOI:** 10.1093/icvts/ivag173

**Published:** 2026-06-24

**Authors:** Jonggeun Lee, Ho Seong Cho, Jeong Su Cho, Yeong Dae Kim, Hyo Yeong Ahn, Sang Hun Kim

**Affiliations:** Department of Thoracic & Cardiovascular Surgery, School of Medicine, Pusan National University, Biomedical Research Institute, Pusan National University Hospital, Busan 49241, Korea; Department of Thoracic & Cardiovascular Surgery, School of Medicine, Kyung Hee University Medical Center, Seoul 02447, Republic of Korea; Department of Thoracic & Cardiovascular Surgery, School of Medicine, Pusan National University, Biomedical Research Institute, Pusan National University Hospital, Busan 49241, Korea; Department of Thoracic & Cardiovascular Surgery, School of Medicine, Pusan National University, Biomedical Research Institute, Pusan National University Hospital, Busan 49241, Korea; Department of Thoracic & Cardiovascular Surgery, School of Medicine, Pusan National University, Biomedical Research Institute, Pusan National University Hospital, Busan 49241, Korea; Department of Thoracic & Cardiovascular Surgery, School of Medicine, Pusan National University, Biomedical Research Institute, Pusan National University Hospital, Busan 49241, Korea; Department of Rehabilitation Medicine, School of Medicine, Pusan National University, Biomedical Research Institute, Pusan National University Hospital, Busan, 49241, Korea

**Keywords:** CPET, NSCLC, ventilatory efficiency, postoperative complication, risk stratification

## Abstract

**Objectives:**

We examined whether cardiopulmonary exercise testing (CPET) variables predict 30‑day postoperative complications in patients undergoing anatomical resection for non–small cell lung cancer (NSCLC).

**Methods:**

Consecutive patients who underwent segmentectomy or greater between January 2023 and March 2025 at a single tertiary centre were reviewed. All patients underwent CPET within 30 days preoperatively. Data on demographics, comorbidities, pulmonary function, operative factors, and outcomes were collected. Associations were assessed using univariable and multivariable logistic regression; discrimination was evaluated with receiver operating characteristic curve (ROC). Results with 2‑sided α = 0.05 were considered significant. Statistical analyses were conducted with R 4.4.2 (stats).

**Results:**

Among 353 patients (mean age 68.4 ± 8.4 years; 58.1% male individuals), 33 (9.4%) experienced complications. Patients were older (71.8 vs 68.0 years) and more often male individuals (81.8% vs 55.6%) than controls; they had lower body mass index (BMI) (23.1 vs 24.4 kg/m^2^) and lower forced expiratory volume in 1 second/forced vital capacity (FEV_1_/FVC) (69.5% vs 72.7%). In the univariable analysis, age (odds ratio [OR] 1.07), female sex (OR 0.28 vs male), BMI (OR 0.88 per kg/m^2^), FEV_1_/FVC (OR 0.96 per %), ventilatory equivalent for carbon dioxide (VE/VCO_2_) slope (OR 1.06 per unit), attained stage (OR 0.66 per stage), and operation time (OR 1.58 per hour) were associated with complications. In the multivariable analysis, BMI (OR 0.86, 95% confidence interval [CI] 0.75-1.00), FEV_1_/FVC (OR 0.94, 95% CI, 0.90-0.99), and VE/VCO_2_ slope (OR 1.06, 95% CI, 1.00-1.11) remained independent predictors. Receiver operating characteristic curves showed poor discrimination: peak oxygen consumption (VO_2_peak) area under the curve (AUC), 0.52; anaerobic threshold (AT), 0.59; VE/VCO_2_ slope, 0.40; and AT time 0.43. Dichotomized cut‑offs were generally non‑informative.

**Conclusions:**

Individual CPET variables had limited discriminative accuracy (AUC < 0.6). Cardiopulmonary exercise testing should complement clinical and spirometric predictors rather than serve as a stand‑alone gatekeeper.

## Graphical abstract

**Figure:**
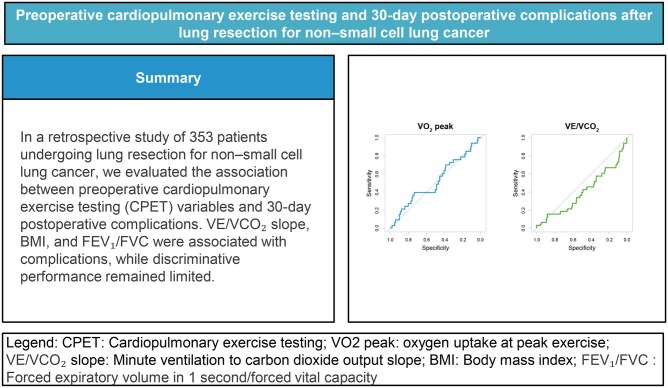

## INTRODUCTION

Lung resection remains the cornerstone of curative treatment for early-stage non–small cell lung cancer (NSCLC). However, pulmonary resection is associated with substantial postoperative morbidity, particularly in patients with limited cardiopulmonary reserve or multiple comorbidities. Accurate preoperative risk stratification is therefore essential to guide surgical decision-making and perioperative management.^1^^,^^2^

Pulmonary function tests such as forced expiratory volume in 1 second (FEV_1_) and diffusion capacity for carbon monoxide (DLCO) have traditionally been used to estimate operative risk. However, these measurements provide only partial information about functional capacity and may not fully reflect a patient’s global cardiopulmonary reserve.^2^^,^^3^ For this reason, cardiopulmonary exercise testing (CPET) has been increasingly incorporated into preoperative evaluation algorithms for lung resection.^1^^,^^2^

Cardiopulmonary exercise testing provides an integrated assessment of cardiovascular, pulmonary, and metabolic responses to exercise. Several CPET-derived parameters—including peak oxygen consumption (VO_2_peak), anaerobic threshold (AT), and the ventilatory equivalent for carbon dioxide slope (VE/VCO_2_ slope)—have been proposed as predictors of postoperative complications and mortality after lung resection.^4–6^ Current international guidelines recommend CPET particularly in patients with borderline pulmonary function to further refine surgical risk assessment.^1^^,^^2^

Despite these recommendations, the clinical utility of specific CPET parameters in contemporary thoracic surgical populations remains debated. Many previous studies were conducted in highly selected high-risk cohorts or focused primarily on severe cardiopulmonary outcomes.^4^^,^^6^ In contrast, modern thoracic surgery increasingly involves minimally invasive approaches and patients with relatively preserved lung function, which may influence the predictive performance of CPET variables.^7^

Therefore, the present study aimed to evaluate the association between preoperative CPET parameters and postoperative complications in patients undergoing lung resection for lung cancer. In addition, we assessed the discriminative ability of selected CPET variables to determine their potential role in contemporary perioperative risk stratification.

## METHODS

### Study design and population

**Figure 1.**
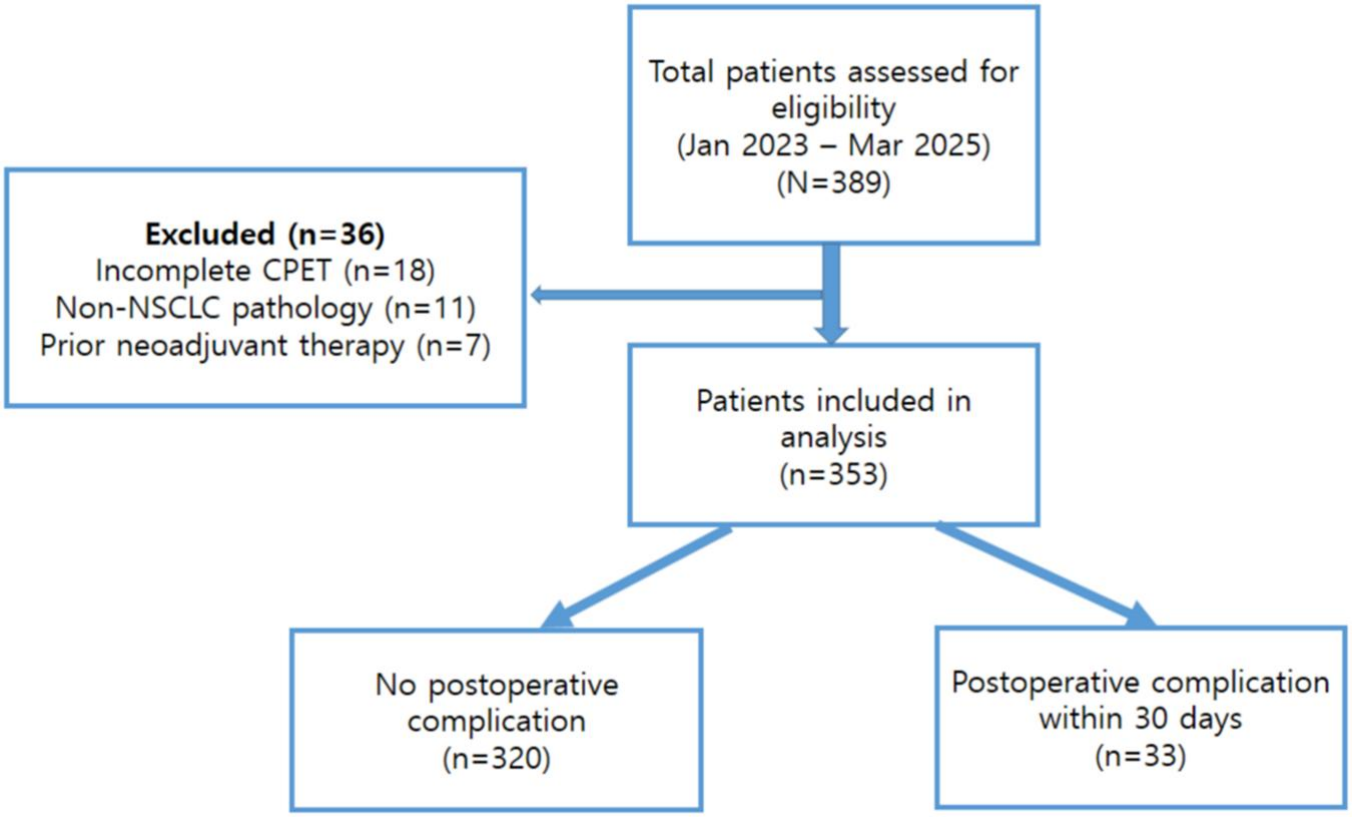
Patient Flow Diagram of the Study Cohort

This single‑centre retrospective study involved adults undergoing segmentectomy or greater for primary NSCLC (January 2023-March 2025). All patients underwent CPET within 30 days preoperatively. The exclusion criteria were as follows: incomplete CPET, non‑NSCLC pathology, and prior neoadjuvant therapy (**Figure 1**).

### Variables and outcomes

Collected variables are summarized in **Table 1**. The primary end-point was any 30‑day postoperative complication (pulmonary, cardiovascular, or intensive care unit [ICU]‑related). Types and frequencies of the postoperative complications are provided in **Table 2-1**.

**Table 1.** Baseline Characteristics of Patients with and without Postoperative Complications

Variable	Overall (*n* = 353)	No complications (*n* = 320)	Complications (*n* = 33)
**Demographics**			
Age, years,	68.37 (8.35)	68.02 (8.49)	71.76 (5.82)
Male sex, *n* (%)	205 (58.1)	178 (55.6)	27 (81.8)
Height, cm	162.16 (8.27)	162.05 (8.31)	163.22 (7.99)
Weight, kg	64.11 (11.32)	64.36 (11.50)	61.70 (9.21)
BMI, kg/m²	24.30 (3.33)	24.42 (3.36)	23.14 (2.84)
Smoking history, *n* (%)			
Non-smoker	190 (53.8)	176 (55.0)	14 (42.4)
Ex-smoker	97 (27.5)	85 (26.6)	12 (36.4)
Current smoker	66 (18.7)	59 (18.4)	7 (21.2)
ECOG performance status, *n* (%)			
0	352 (99.7)	319 (99.7)	33 (100.0)
1	1 (0.3)	1 (0.3)	0 (0.0)
**Comorbidities, *n* (%)**			
Underlying lung disease (COPD, emphysema)	53 (15.0)	48 (15.0)	5 (15.2)
Hypertension	173 (49.0)	156 (48.8)	17 (51.5)
Diabetes mellitus	88 (24.9)	80 (25.0)	8 (24.2)
Previous tuberculosis	35 (9.9)	32 (10.0)	3 (9.1)
Neurologic event	21 (5.9)	20 (6.2)	1 (3.0)
Other malignancy	47 (13.3)	43 (13.4)	4 (12.1)
Liver disease	16 (4.5)	16 (5.0)	0 (0.0)
**Pulmonary function**			
FEV_1_, L	2.35 (0.55)	2.36 (0.56)	2.23 (0.39)
FEV_1_, % predicted	87.20 (15.60)	87.63 (15.54)	83.06 (15.84)
FVC, L	3.25 (0.72)	3.25 (0.73)	3.24 (0.59)
FVC, % predicted	87.12 (13.36)	87.52 (13.42)	83.30 (12.30)
FEV_1_/FVC, %	72.37 (7.87)	72.67 (7.77)	69.48 (8.38)
DLCO, % predicted	88.30 (16.44)	88.67 (16.25)	84.76 (18.03)
**CPET parameters**			
Peak RER	1.09 (0.09)	1.10 (0.09)	1.07 (0.06)
VO₂ peak (mL/kg/min)	24.40 (5.41)	24.37 (5.41)	24.77 (5.49)
Maximal METs	6.95 (1.56)	6.94 (1.56)	7.05 (1.58)
Anaerobic threshold (mL/kg/min), mean (SD)	17.82 (4.12)	17.70 (4.08)	19.00 (4.40)
AT time, s	760.43 (308.57)	766.89 (307.37)	697.82 (318.00)
VE/VCO₂ slope	30.17 (6.59)	29.88 (6.04)	33.00 (10.23)
**Operative and postoperative outcomes**			
Surgical approach (VATS/robot/open)	280/70/3	253/64/3	27/6/0
Operation time, h	2.49 (0.93)	2.44 (0.88)	2.94 (1.26)
ICU stay, days	1.34 (3.46)	1.03 (0.18)	4.39 (11.00)
ICU stay ≥2 days, n(%)	16 (4.5)	10 (3.1)	6 (18.2)
Hospital stay, days	6.37 (5.72)	5.30 (2.27)	16.70 (13.66)

Values are presented as mean (standard deviation) unless otherwise indicated.

Abbreviations: AT, anaerobic threshold; BMI, body mass index (kg/m^2^); COPD, chronic obstructive pulmonary disease; DLCO, carbon monoxide diffusion capacity; ECOG, Eastern Cooperative Oncology Group; FEV_1_, forced expiratory volume in 1 s; FVC, forced vital capacity; ICU, intensive care unit; MET, metabolic equivalent; Peak RER, peak respiratory exchange ratio; TB, tuberculosis; VCO_2_, carbon dioxide excretion at peak exercise; VE, ventilation at peak exercise; VO_2_peak, oxygen uptake at peak exercise.

**Table 2-1.** Postoperative Complications and Severity according to Clavien-Dindo Classification

**Variable**	*N* = 33 (%)
Prolonged air leak	15 (45.5)
Pneumonia	8 (24.2)
Delirium tremens	7 (21.2)
ARDS	4 (12.1)
Death	4 (12.1)
Tracheostomy	3 (9.1)
Chylothorax	2 (6.1)
Vocal cord palsy	2 (6.1)
New renal failure	2 (6.1)

Abbreviation: ARDS, acute respiratory distress syndrome.

Each postoperative complication was graded individually according to the treatment required and clinical severity using the Clavien-Dindo classification. When multiple complications occurred in the same patient, the highest Clavien-Dindo grade was used for patient-level severity analysis.

### Statistics

Continuous variables were subjected to independent *t*-test or a Wilcoxon rank sum test depending on whether normality is satisfied. For categorical variables, a chi-squared test or Fisher’s exact test was performed. Logistic regression yielded odds ratios (ORs) with 95% confidence intervals (CIs). Variables for multivariable logistic regression were selected based on clinical relevance and prior literature rather than univariate significance. Age, sex, body mass index (BMI), FEV_1_/forced vital capacity (FVC), and VE/VCO_2_ slope were included a priori as potential confounders reflecting overall fitness, ventilatory reserve, and anthropometric risk.

Additional variables (operation time and clinical stage) were incorporated according to their established association with postoperative outcomes in thoracic surgery. No automated stepwise or data-driven selection methods were used.

Model calibration and discrimination were verified using goodness-of-fit tests and area under the receiver operating characteristic curve (AUC) analysis. Results with 2-sided α = 0.05 were considered statistically significant. All statistical analyses were performed using R Statistical Software (version 4.4.2; R Core Team, 2024).

## RESULTS

### Baseline characteristics

Among 353 patients, 33 (9.4%) had complications. Affected patients were older (71.76 vs 68.02 years) and more often male individuals (81.8% vs 55.6%), and had a lower BMI (23.14 vs 24.42 kg/m^2^) and lower FEV_1_/FVC than those without complications (69.48% vs 72.67%). Other demographics, comorbidities, and pulmonary function measures were similar between them (**Table 1**). Postoperative complications were further graded according to the Clavien-Dindo classification. Most complications were low-to-moderate severity (grade I-III), whereas major complications (grade ≥IV) were less frequent (**Table 2-2**).

**Table 2-2.** Distribution of Complication Severity

Clavien-Dindo grade	*N* = 33 (%)
Grade I	0 (0)
Grade II	17 (51.5)
Grade IIIa	20 (60.6)
Grade IIIb	0 (0)
Grade IVa	6 (18.2)
Grade IVb	0 (0)
Grade V	4 (12.1)

Grade I: Any deviation from the normal postoperative course without the need for pharmacological treatment or surgical, endoscopic, and radiological interventions.

Grade II: Requiring pharmacological treatment with drugs other than such allowed for Grade I complications. Blood transfusions and total parenteral nutrition (TPN) are also included.

Grade IIIa: Requiring surgical, endoscopic, or radiological intervention. Intervention not under general anaesthesia.

Grade IIIb: Intervention not under general anaesthesia.

Grade IVa: Life-threatening complication (including those affecting the brain) requiring intensive care management. Single organ dysfunction (including dialysis).

Grade IVb: Multi organ dysfunction.

Grade V: Death of a patient.

### Postoperative complications according to extent of resection

When postoperative complications were stratified according to the extent of lung resection (segmentectomy, lobectomy, and bilobectomy), no statistically significant differences were observed in the incidence of individual complications across surgical categories (all *P* > .05, Fisher’s exact test). Specifically, rates of cardiorespiratory complications, prolonged air leak, delirium, and 30-day mortality were comparable between groups (**Table S2**).

### Subgroup analysis of cardiopulmonary complications

To address the potential heterogeneity of postoperative complications, a secondary descriptive analysis focusing on cardiopulmonary complications was performed (**Table S3**). Cardiopulmonary complications were defined as pneumonia, acute respiratory distress syndrome (ARDS), respiratory failure requiring tracheostomy, and death.

A total of 19 cardiopulmonary complications were identified; however, some patients experienced multiple complications. In this subgroup, CPET variables demonstrated a similar directional pattern to the overall findings in an exploratory assessment (**Table S4**). In particular, VE/VCO_2_ slope remained positively associated with increased risk of cardiopulmonary complications, although statistical significance was not consistently observed, likely due to the limited number of events and reduced statistical power.

### Univariable and multivariable analyses

Univariate analysis results (**Table 3**) showed associations of age (OR 1.07, *P* = .014), sex (female vs male OR 0.28, *P* = .006), BMI (OR 0.88 per kg·m^−2^, *P* = .037), FEV_1_/FVC (OR 0.96 per %, *P* = .029), VE/VCO_2_ slope (OR 1.06 per unit, *P* = .013), attained stage (OR 0.66 per stage, *P* = .047), and operation time (OR 1.58 per hour, *P* = .005). In the multivariable model (**Table 4**), which included age, sex, BMI, FEV_1_/FVC, VE/VCO_2_ slope, operation time, and pathological stage as predefined clinically relevant variables, BMI (OR 0.86, 95% CI, 0.75-1.00, *P* = .048), FEV_1_/FVC (OR 0.94, 95% CI, 0.90-0.99, *P* = .019), and VE/VCO_2_ slope (OR 1.06, 95% CI, 1.00-1.11, *P* = .040) remained independent predictors of 30-day postoperative complications.

**Table 3.** Univariate Analysis of Significant Risk Factors for Postoperative Complications

Variable	No complications (*n* = 320)	Complications (*n* = 33)	OR [95% CI]	*P*-value
Age, years (mean ± SD)	68.02 (8.49)	71.76 (5.82)	1.07 [1.01, 1.13]	.014
Sex, male, *n* (%)	178 (55.6)	27 (81.8)	Ref.	
Sex, female, *n* (%)	142 (44.4)	6 (18.2)	0.28 [0.11, 0.69]	.006
BMI, kg/m² (mean ± SD)	24.42 (3.36)	23.14 (2.84)	0.88 [0.79, 0.99]	.037
FEV_1_/FVC (%)	72.67 (7.77)	69.48 (8.38)	0.96 [0.91, 0.99]	.029
AT (mL/kg/min), mean (SD)	17.70 (4.08)	19.00 (4.40)	1.08 [0.99, 1.18]	.085
AT time, s	766.89 (307.37)	697.82 (318.00)	0.99 [0.99, 1.00]	.222
VE/VCO_2_ slope	29.88 (6.04)	33.00 (10.23)	1.06 [1.01, 1.11]	.013
Attained stage	4.48 (0.78)	4.18 (1.07)	0.66 [0.44, 0.99]	.047
Operation time, h	2.44 (0.88)	2.94 (1.26)	1.58 [1.15, 2.18]	.005
ICU stay ≥2 days, *n* (%)	10 (3.1)	6 (18.2)	6.89 [2.33, 20.40]	<.001
Hospital stay, days	5.30 (2.27)	16.70 (13.66)	1.48 [1.32, 1.66]	<.001

Abbreviations: AT, anaerobic threshold; BMI, body mass index; CI, confidence interval; FEV_1_, forced expiratory volume in 1 s; FVC, forced vital capacity; ICU, intensive care unit; OR, odds ratio.

**Table 4.** Multivariable Logistic Regression for Independent Predictors

Variable	No complications (*n* = 320)	Complications (*n *= 33)	OR [95% CI]	*P*-value
BMI (mean (SD))	24.42 (3.36)	23.14 (2.84)	0.86 [0.75, 0.99]	.043
FEV_1_/FVC (%)	72.67 (7.77)	69.48 (8.38)	0.94 [0.89, 0.99]	.022
Preop DLCO, % predicted	88.67 (16.25)	84.76 (18.03)	1.01 [0.98, 1.03]	.703
VO₂ peak (mL/kg/min)	24.37 (5.41)	24.77 (5.49)	1.04 [0.80, 1.35]	.756
Anaerobic threshold	21.82 (73.53)	19.00 (4.40)	1.00 [0.98, 1.01]	.948
VE/VCO_2_ slope	29.88 (6.04)	33.00 (10.23)	1.06 [1.00, 1.11]	.035

Abbreviations: BMI, body mass index; CI, confidence interval; DLCO, diffusion capacity for carbon monoxide; FEV_1_, forced expiratory volume in 1 s; FVC, forced vital capacity; OR, odds ratio.

### ROC curve of CPET performance

**Figure 2.**
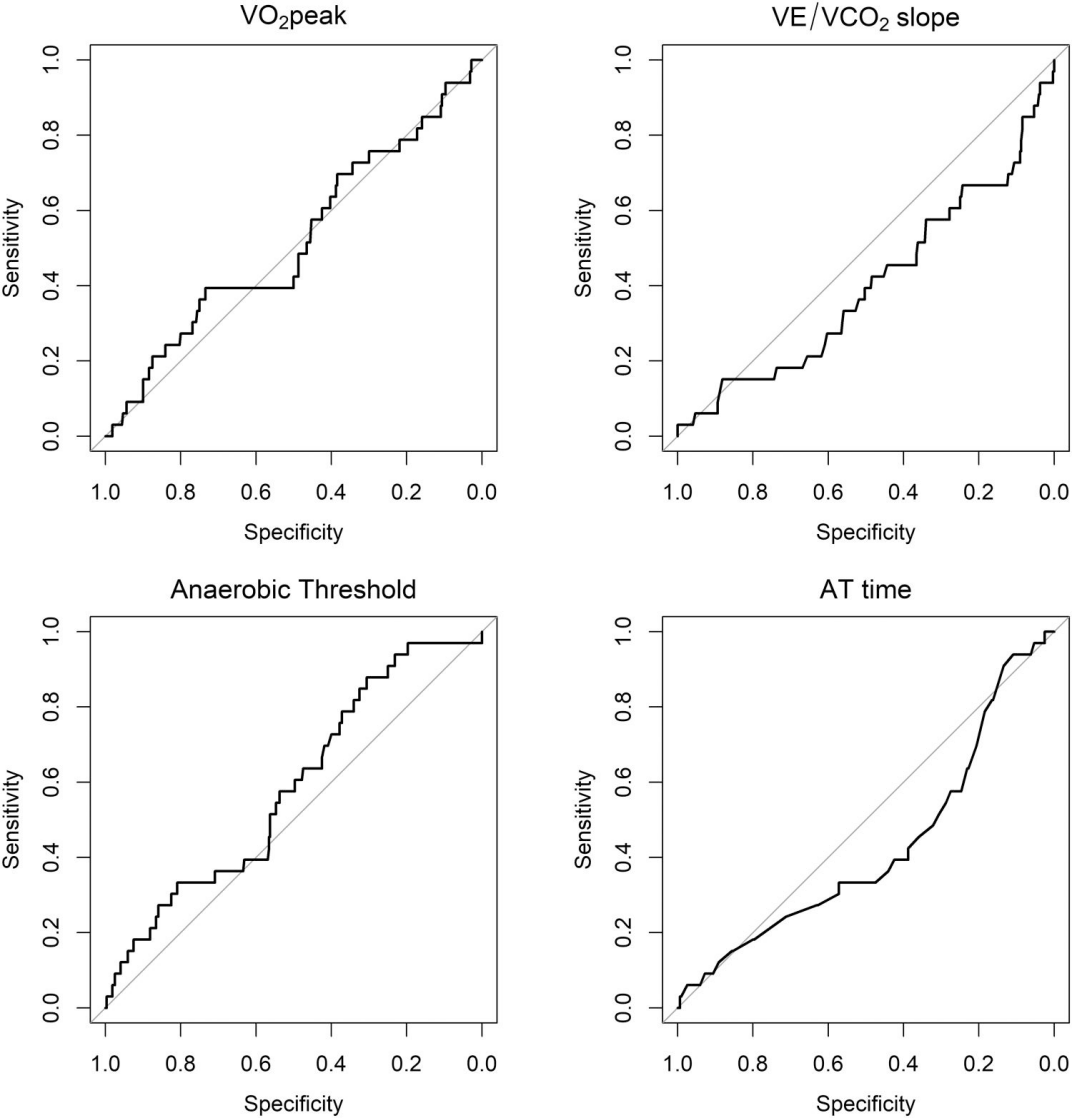
ROC Curves for CPET Variables. AUCs: VO_2_peak 0.52; VE/VCO_2_ slope 0.40; anaerobic threshold 0.59; AT time 0.43. Receiver-operating characteristic (ROC) curves for preoperative CPET variables in predicting 30-day postoperative complications after anatomical lung resection. AUCs: VO_2_peak, 0.52; VE/VCO_2_ slope, 0.40; anaerobic threshold (AT), 0.59; and time-to-AT, 0.43. The diagonal line denotes no-discrimination; among the metrics, AT showed the highest (though modest) discrimination, whereas the VE/VCO_2_ slope and time-to-AT performed poorly in this cohort

Receiver operating characteristic curves (**Figure 2**) demonstrated limited discrimination for individual CPET variables: VO_2_peak AUC, 0.52; AT, 0.59; VE/VCO_2_ slope, 0.40; and AT time, 0.43. All AUC values were <0.60.

### Dichotomized CPET cut‑offs

**Figure 3.**
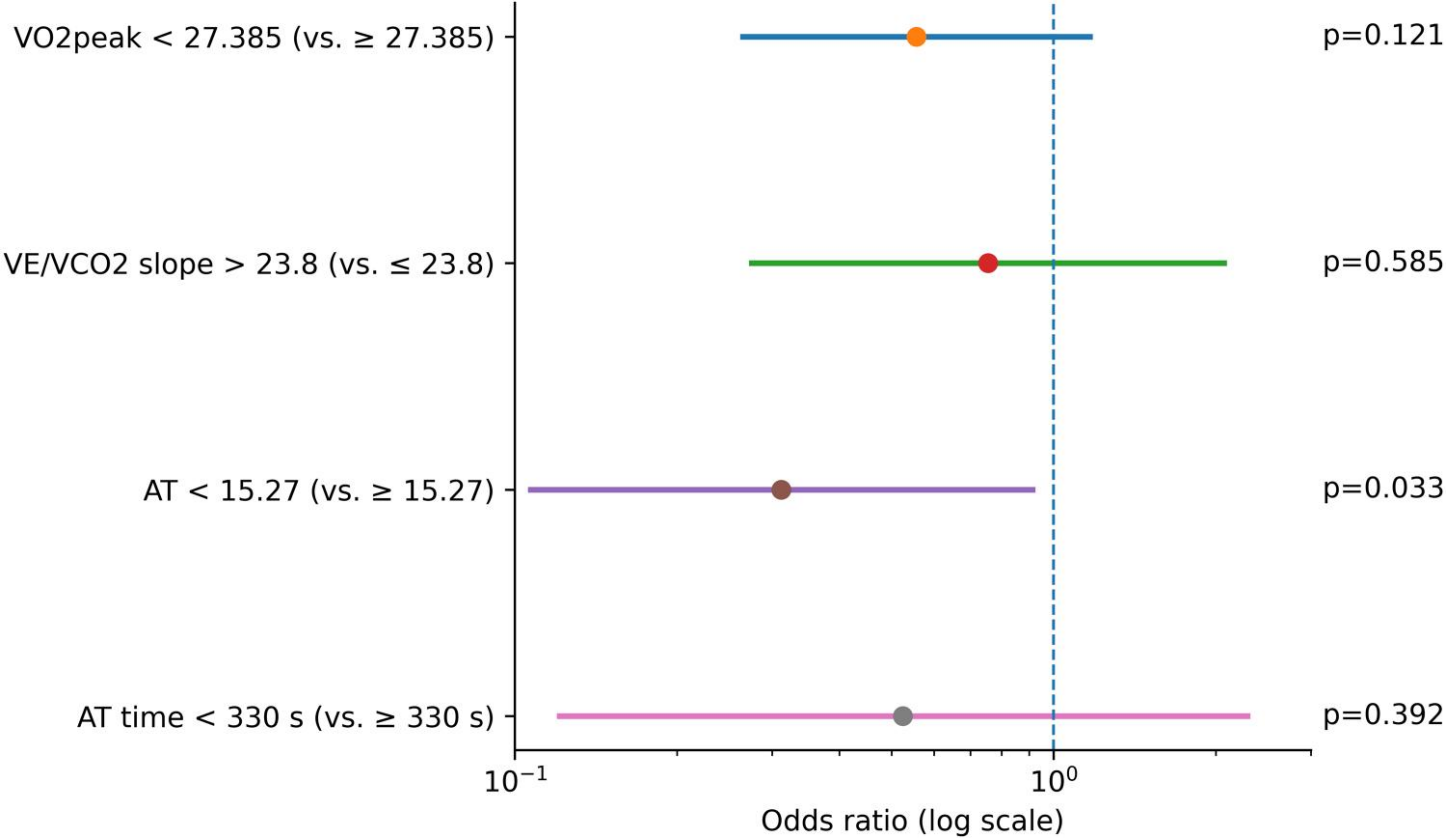
Univariate Odds Ratios (95% CI) for Postoperative Complications according to Dichotomized Cardiopulmonary Exercise Testing (CPET) Cutoff Values, Displayed as a Forest Plot. Odds ratios (dots) and 95% confidence intervals (bars) are shown on a log scale. Reference categories: VO_2_peak, ≥27.385 mL·kg^−1^·min^−1^; VE/VCO_2_ slope, ≤23.8; AT, ≥15.27 mL·kg^−1^·min^−1^; AT time, ≥330 s. OR>1 indicates higher odds of complications relative to the reference. Abbreviations: AT, anaerobic threshold; CI, confidence interval; CPET, cardiopulmonary exercise testing

When dichotomized by data‑driven cut‑offs empirically derived median split (**Figure 3**), the CPET variables were not predictive: VO_2_peak, <27.385 vs ≥27.385 (13/98 vs 20/255, OR 0.56, *P* = .121); VE/VCO_2_ slope, >23.8 vs ≤23.8 (28/310 vs 5/43, OR 0.76, *P* = .585); and AT time, <330 s vs ≥330 s (2/37 vs 31/316, OR 0.53, *P* = .392). The apparent lower OR for AT <15.27 vs ≥15.27 (4/101 vs 29/252, OR 0.32, *P* = .036) likely reflects sparse events and selection, rather than a robust discriminative effect.

## DISCUSSION

Across a contemporary surgical cohort, the VE/VCO_2_ slope retained a statistical association with postoperative complications and remained significant after adjustment for BMI and FEV_1_/FVC. This observation is broadly consistent with previous reports demonstrating an association between ventilatory inefficiency and adverse postoperative outcomes following pulmonary resection.^4–7^ In particular, increased ventilatory inefficiency during exercise, reflected by an elevated VE/VCO_2_ slope, has been linked with increased risk of pulmonary complications and postoperative mortality in patients undergoing lung cancer surgery.^4–6^

From a clinical perspective, a 5-unit increase in VE/VCO_2_ slope corresponded to approximately 30% higher odds of postoperative complications. This finding supports the physiologic plausibility of ventilatory inefficiency as a marker of impaired cardiopulmonary reserve and perioperative vulnerability. Ventilatory inefficiency during exercise may reflect abnormalities in ventilation-perfusion matching, pulmonary vascular reserve, or cardiovascular performance, all of which may contribute to adverse perioperative outcomes.^6^^,^^8^

Importantly, the multivariable model was deliberately constructed using clinically meaningful covariates defined a priori rather than relying solely on univariate statistical thresholds. This approach is commonly recommended in clinical research to reduce model instability and the risk of overfitting, particularly in studies with relatively limited numbers of outcome events. By incorporating clinically relevant covariates into the model, the present analysis aimed to estimate independent associations while preserving clinical interpretability.

Despite this statistical association, the stand-alone discriminative performance of individual CPET variables was limited, with area-under-the-curve values below 0.60. This apparent discrepancy highlights the important distinction between statistical association and clinical discrimination. Variables may retain independent associations with outcomes yet fail to meaningfully separate individuals at the bedside. Similar findings have been reported in contemporary surgical cohorts in which physiologic variables demonstrate limited predictive performance when evaluated in relatively low-risk populations.^7^^,^^9^

Several factors may explain the modest discriminative performance observed in the present study. First, modern thoracic surgical populations increasingly consist of patients with earlier-stage disease and relatively preserved cardiopulmonary function. Furthermore, advances in minimally invasive surgical techniques and perioperative management have contributed to improved postoperative outcomes and reduced complication rates compared with historical cohorts.^2^ Consequently, the gradient of physiologic risk within contemporary surgical populations may be attenuated. These findings suggest that while CPET variables may reflect physiologic vulnerability, their discriminative performance remains modest in contemporary low-risk surgical populations.

The inclusion of heterogeneous postoperative complications represents an important limitation when interpreting the predictive value of CPET variables. Several complications included in the primary end-point, such as prolonged air leak, vocal cord palsy, and chylothorax, are more closely related to surgical technique or intraoperative factors rather than preoperative cardiopulmonary reserve. These findings highlight that the inclusion of heterogeneous end-points may attenuate the observed predictive value of CPET variables.

To address this, we performed a secondary descriptive analysis focusing specifically on cardiopulmonary complications. In this subgroup, the direction of association between CPET variables and outcomes remained consistent with the primary analysis, although statistical significance was limited due to the small number of events, and these findings should be interpreted as exploratory.

Second, the absence of significant differences in complication rates according to the extent of resection further illustrates the highly selected nature of contemporary surgical cohorts. Patients undergoing more extensive resections are typically subjected to rigorous preoperative functional evaluation and multidisciplinary assessment, which may mitigate the observable impact of physiologic risk factors on postoperative outcomes.^8^^,^^10^ This selection effect, combined with the relatively low overall event rate in the present cohort, plausibly explains the limited discriminative performance of CPET metrics observed in this study.

Consistent with this interpretation, no significant differences were observed in the incidence of individual complications, including cardiopulmonary events, prolonged air leak, delirium, or 30-day mortality. These findings likely reflect careful preoperative patient selection and further support the notion that contemporary thoracic surgical cohorts represent a functionally optimized population in whom traditional physiologic risk gradients may be less pronounced.^3^^,^^11^

Although AT demonstrated the highest AUC among the evaluated CPET variables, neither its mean value nor the time to threshold differed significantly between groups. Furthermore, dichotomization of CPET variables using empirically derived cutoffs did not improve predictive performance and produced unstable signals, particularly for AT. These findings highlight the limitations of threshold-based interpretation in relatively low-event surgical populations and support previous recommendations that CPET parameters should be interpreted within a broader clinical context rather than used as isolated predictors.^1^^,^^8^

Current international guidelines recommend CPET primarily as an adjunctive tool in patients with borderline pulmonary function or discordant spirometric findings.^1^^,^^10^ Within this framework, CPET parameters should complement rather than replace established clinical and spirometric predictors during preoperative risk assessment. Taken together, these findings suggest that CPET—particularly the VE/VCO_2_ slope—may provide useful physiologic information but should be integrated with clinical and pulmonary function variables rather than used as a stand-alone triage tool.

Beyond risk stratification, CPET may also play a role in perioperative optimization strategies. Increasing attention has been directed towards prehabilitation programs aimed at improving functional capacity before surgery. Exercise-based prehabilitation, often combined with nutritional and behavioural interventions, has been associated with improvements in functional capacity and postoperative recovery in patients undergoing lung cancer surgery.^8^^,^^10^^,^^12–16^ In this context, CPET may serve not only as a risk assessment tool but also as a means of identifying patients who could benefit most from targeted preoperative optimization.

Future prospective studies integrating CPET parameters into multicomponent predictive models and validating them externally are warranted to clarify their additive value in surgical risk stratification. The retrospective single-centre design of the present study, the limited event rate (9.4%), and the absence of external validation may have attenuated the apparent discrimination and precision of the estimates. Nevertheless, the present findings contribute to refining the real-world applicability of CPET in contemporary thoracic surgical practice.

### Limitations

Several limitations of this study should be acknowledged.

First, this was a retrospective single-centre study, which may introduce selection bias and limit the generalizability of the findings. Although all consecutive patients meeting the inclusion criteria were analysed, the retrospective design inherently restricts control over potential confounding variables.

Second, the number of postoperative events was relatively small, which may limit the statistical power of the analyses. With a limited number of complications, particularly major complications, the ability to detect statistically significant associations may be reduced. Consequently, the findings should be interpreted with caution and considered primarily hypothesis-generating.

Third, the study population consisted predominantly of patients with relatively preserved pulmonary function and a low overall complication rate. This relatively low-risk cohort may partly explain the modest discriminative performance of the evaluated CPET parameters.

Fourth, postoperative complications were heterogeneous in nature and included both technical and cardiopulmonary events. Although all complications were systematically graded according to the Clavien-Dindo classification, this heterogeneity may influence the strength of associations between CPET parameters and postoperative outcomes.

Finally, external validation in larger multicentre cohorts would be necessary to confirm the generalizability and clinical utility of the findings.

## CONCLUSION

Ventilatory equivalent for carbon dioxide (VE/VCO_2_) slope, BMI, and FEV_1_/FVC were independently associated with 30-day postoperative complications; however, individual CPET variables showed limited discriminative accuracy. Cardiopulmonary exercise testing should therefore be considered an adjunct to clinical and spirometric assessment rather than a stand-alone gatekeeper in surgical candidates with NSCLC. Given the relatively small number of outcome events and the low-risk surgical population included in this study, these results should be interpreted cautiously. Further large-scale prospective studies are required to better define the role of CPET parameters in contemporary thoracic surgical risk stratification.

## Supplementary Material

ivag173_Supplementary_Data

## Data Availability

The raw data for this study can be obtained from the corresponding author upon a reasonable request.

## STATISTICS

Two‑sided tests with α = 0.05; analyses in R 4.4.2 (R Core Team, 2024) using the stats package.
