# Supplementary material for: Preoperative Cardiopulmonary Exercise Testing and 30-Day Postoperative Complications After Lung Resection for Non–Small Cell Lung Cancer: A Retrospective Cohort Study
Source: Interdiscip Cardiovasc Thorac Surg. 2026 Jun 24;41(7):ivag173. doi: 10.1093/icvts/ivag173 (PMC13345927; doi:10.1093/icvts/ivag173)
Supplement: ivag173_Supplementary_Data [file ivag173_supplementary_data.docx]

## Supplementary Table 1. Full univariate analysis of risk factors

| Variable | No complications (n=320) | Complications (n=33) | OR [95% CI] | p-value |
| --- | --- | --- | --- | --- |
| Age, years  (mean ± SD) | 68.02 (8.49) | 71.76 (5.82) | 1.068  [1.014, 1.125] | 0.014 |
| Sex, Male n (%) | 178 (55.6) | 27 (81.8) | Ref. |  |
| Sex, Female  n (%) | 142 (44.4) | 6 (18.2) | 0.279  [0.112, 0.693] | 0.006 |
| BMI, kg/m²  (mean ± SD) | 24.42 (3.36) | 23.14 (2.84) | 0.883  [0.785, 0.992] | 0.037 |
| FEV1/FVC (%) | 72.67 (7.77) | 69.48 (8.38) | 0.955  [0.916, 0.995] | 0.029 |
| VE/VCO_2_ slope | 29.88 (6.04) | 33.00 (10.23) | 1.058  [1.012, 1.106] | 0.013 |
| Attained stage | 4.48 (0.78) | 4.18 (1.07) | 0.657  [0.435, 0.994] | 0.047 |
| Operation time, h | 2.44 (0.88) | 2.94 (1.26) | 1.582  [1.147, 2.182] | 0.005 |
| ICU stay  ≥2 days, n (%) | 10 (3.1) | 6 (18.2) | 6.889  [2.326, 20.404] | <0.001 |
| Hospital stay,  days | 5.30 (2.27) | 16.70 (13.66) | 1.482  [1.323, 1.660] | <0.001 |
| Height, cm  (mean ± SD) | 162.05 (8.31) | 163.22 (7.99) | 1.017  [0.974, 1.062] | 0.438 |
| Weight, kg  (mean ± SD) | 64.36 (11.50) | 61.70 (9.21) | 0.978  [0.946, 1.012] | 0.200 |
| Smoking history, non-smoker | 176 (55.0) | 14 (42.4) | Ref. |  |
| Smoking history, ex-smoker | 85 (26.6) | 12 (36.4) | 1.775  [0.787, 4.003] | 0.167 |
| Smoking history, current smoker | 59 (18.4) | 7 (21.2) | 1.492  [0.575, 3.872] | 0.411 |
| Underlying lung disease | 283 (88.4) | 30 (90.9) | Ref. |  |
| Yes | 37 (11.6) | 3 (9.1) | 0.765  [0.222, 2.631] | 0.671 |
| Hypertension | 164 (51.2) | 16 (48.5) | Ref. |  |
| Yes | 156 (48.8) | 17 (51.5) | 1.117  [0.545, 2.288] | 0.762 |
| Cardiovascular disease, PA | 32 (10.0) | 4 (12.1) | 1.315  [0.433, 3.997] | 0.629 |
| Cardiovascular disease, MI | 4 (1.2) | 2 (6.1) | 5.259  [0.921, 30.044] | 0.062 |
| Diabetes mellitus | 240 (75.0) | 25 (75.8) | Ref. |  |
| Yes | 80 (25.0) | 8 (24.2) | 0.960  [0.416, 2.213] | 0.924 |
| Tuberculosis history | 288 (90.0) | 30 (90.9) | Ref. |  |
| Yes | 32 (10.0) | 3 (9.1) | 0.900  [0.260, 3.115] | 0.868 |
| Neurologic event, stroke | 13 (4.1) | 1 (3.0) | 0.721  [0.091, 5.694] | 0.757 |
| Neurologic event, other | 7 (2.2) | 0 (0.0) | 0.000  [0.000, Inf] | 0.987 |
| Other malignancy | 277 (86.6) | 29 (87.9) | Ref. |  |
| Yes | 43 (13.4) | 4 (12.1) | 0.889  [0.298, 2.652] | 0.832 |
| Other comorbidity | 39 (12.2) | 3 (9.1) | Ref. |  |
| Yes | 281 (87.8) | 30 (90.9) | 1.388  [0.404, 4.763] | 0.602 |
| Preop FEV1, L | 2.36 (0.56) | 2.23 (0.39) | 0.644  [0.320, 1.296] | 0.218 |
| Preop FEV1, % predicted | 87.63 (15.54) | 83.06 (15.84) | 0.982  [0.961, 1.004] | 0.110 |
| Preop FVC, L | 3.25 (0.73) | 3.24 (0.59) | 0.989  [0.600, 1.630] | 0.964 |
| Preop FVC, % predicted | 87.52 (13.42) | 83.30 (12.30) | 0.978  [0.953, 1.003] | 0.087 |
| Preop DLCO, % predicted | 88.67 (16.25) | 84.76 (18.03) | 0.985  [0.964, 1.008] | 0.194 |
| Peak RER | 1.10 (0.09) | 1.07 (0.06) | 0.019  [0.000, 1.391] | 0.070 |
| VO_2_peak | 24.37 (5.41) | 24.77 (5.49) | 1.014  [0.949, 1.082] | 0.686 |
| Maximal METs | 6.94 (1.56) | 7.05 (1.58) | 1.044  [0.832, 1.311] | 0.709 |
| % predicted VO_2_peak | 96.00 (18.95) | 93.39 (21.20) | 0.993  [0.974, 1.012] | 0.457 |
| Maximal VO_2_ | 25730.72 (6938.77) | 26669.97 (6362.00) | 1.000  [1.000, 1.000] | 0.456 |
| Maximal RPE | 7.05 (1.96) | 7.33 (2.65) | 1.059  [0.911, 1.231] | 0.453 |
| Anaerobic threshold | 17.70 (4.08) | 19.00 (4.40) | 0.312  [0.107, 0.913] | 0.033 |
| AT time (s) | 866.89 (307.37) | 697.82 (318.00) | 0.525  [0.121, 2.290] | 0.392 |
| Approach VATS | 253 (79.1) | 27 (81.8) | Ref. |  |
| Open | 3 (0.9) | 0 (0.0) | 0.000  [0.000, Inf] | 0.987 |
| Robot | 64 (20.0) | 6 (18.2) | 0.878  [0.348, 2.218] | 0.784 |
| EBL <100 | 115 (36.1) | 7 (21.2) | Ref. |  |
| EBL 100–150 | 135 (42.3) | 18 (54.5) | 2.190  [0.884, 5.430] | 0.090 |
| EBL >150 | 69 (21.6) | 8 (24.2) | 1.905  [0.662, 5.483] | 0.232 |
| ICU stay, days | 1.03 (0.18) | 4.39 (11.00) | 3.477  [0.914, 13.231] | 0.068 |
| Pain VAS POD | 5.73 (1.43) | 5.88 (1.45) | 1.081  [0.824, 1.418] | 0.573 |

**Supplementary Table 2. Postoperative complications stratified by extent of lung resection.**

|  | level | Overall | op_extent = segmentectomy | op_extent = lobectomy | op_extent = bilobectomy |
| --- | --- | --- | --- | --- | --- |
| n |  | 353 | 36 | 315 | 2 |
| prolonged_air_leak (%) | ≤5days | 338 (95.8) | 36 (100.0) | 300 (95.2) | 2 (100.0) |
|  | >5days | 15 (4.2) | 0 (0.0) | 15 (4.8) | 0 (0.0) |
| atelectasis_required_BFS (%) | No | 352 (99.7) | 36 (100.0) | 314 (99.7) | 2 (100.0) |
|  | Yes | 1 (0.3) | 0 (0.0) | 1 (0.3) | 0 (0.0) |
| pneumonia (%) | No | 345 (97.7) | 36 (100.0) | 307 (97.5) | 2 (100.0) |
|  | Yes | 8 (2.3) | 0 (0.0) | 8 (2.5) | 0 (0.0) |
| ALI (%) | No | 353 (100.0) | 36 (100.0) | 315 (100.0) | 2 (100.0) |
|  | Yes | 0 (0.0) | 0 (0.0) | 0 (0.0) | 0 (0.0) |
| ARDS (%) | No | 349 (98.9) | 36 (100.0) | 311 (98.7) | 2 (100.0) |
|  | Yes | 4 (1.1) | 0 (0.0) | 4 (1.3) | 0 (0.0) |
| BPF (%) | No | 353 (100.0) | 36 (100.0) | 315 (100.0) | 2 (100.0) |
|  | Yes | 0 (0.0) | 0 (0.0) | 0 (0.0) | 0 (0.0) |
| pulmonary_embolism (%) | No | 353 (100.0) | 36 (100.0) | 315 (100.0) | 2 (100.0) |
|  | Yes | 0 (0.0) | 0 (0.0) | 0 (0.0) | 0 (0.0) |
| pulmonary_edema (%) | No | 352 (100.0) | 35 (100.0) | 315 (100.0) | 2 (100.0) |
|  | Yes | 0 (0.0) | 0 (0.0) | 0 (0.0) | 0 (0.0) |
| AMI (%) | No | 353 (100.0) | 36 (100.0) | 315 (100.0) | 2 (100.0) |
|  | Yes | 0 (0.0) | 0 (0.0) | 0 (0.0) | 0 (0.0) |
| new_central_neurologic_event (%) | No | 352 (99.7) | 36 (100.0) | 314 (99.7) | 2 (100.0) |
|  | Yes | 1 (0.3) | 0 (0.0) | 1 (0.3) | 0 (0.0) |
| Delirium_tremens (%) | No | 346 (98.0) | 34 (94.4) | 310 (98.4) | 2 (100.0) |
|  | Yes | 7 (2.0) | 2 (5.6) | 5 (1.6) | 0 (0.0) |
| GI_trouble (%) | No | 353 (100.0) | 36 (100.0) | 315 (100.0) | 2 (100.0) |
|  | Yes | 0 (0.0) | 0 (0.0) | 0 (0.0) | 0 (0.0) |
| postop_bleeding_req_reop (%) | No | 352 (99.7) | 36 (100.0) | 314 (99.7) | 2 (100.0) |
|  | Yes | 1 (0.3) | 0 (0.0) | 1 (0.3) | 0 (0.0) |
| UTI (%) | No | 353 (100.0) | 36 (100.0) | 315 (100.0) | 2 (100.0) |
|  | Yes | 0 (0.0) | 0 (0.0) | 0 (0.0) | 0 (0.0) |
| empyema_req_Tx (%) | No | 353 (100.0) | 36 (100.0) | 315 (100.0) | 2 (100.0) |
|  | Yes | 0 (0.0) | 0 (0.0) | 0 (0.0) | 0 (0.0) |
| chylothorax (%) | No | 351 (99.4) | 35 (97.2) | 314 (99.7) | 2 (100.0) |
|  | Yes | 2 (0.6) | 1 (2.8) | 1 (0.3) | 0 (0.0) |
| Tx_modality_for_chylothorax_text (%) |  | 353 (100.0) | 36 (100.0) | 315 (100.0) | 2 (100.0) |
| vocal_cord_palsy (%) | No | 351 (99.4) | 36 (100.0) | 313 (99.4) | 2 (100.0) |
|  | Yes | 2 (0.6) | 0 (0.0) | 2 (0.6) | 0 (0.0) |
| wound_infection (%) | No | 353 (100.0) | 36 (100.0) | 315 (100.0) | 2 (100.0) |
|  | Yes | 0 (0.0) | 0 (0.0) | 0 (0.0) | 0 (0.0) |
| sepsis (%) | No | 353 (100.0) | 36 (100.0) | 315 (100.0) | 2 (100.0) |
|  | Yes | 0 (0.0) | 0 (0.0) | 0 (0.0) | 0 (0.0) |
| new_renal_failure (%) | No | 351 (99.4) | 36 (100.0) | 313 (99.4) | 2 (100.0) |
|  | Yes | 2 (0.6) | 0 (0.0) | 2 (0.6) | 0 (0.0) |
| reintubation (%) | No | 352 (99.7) | 36 (100.0) | 314 (99.7) | 2 (100.0) |
|  | Yes | 1 (0.3) | 0 (0.0) | 1 (0.3) | 0 (0.0) |
| tracheostomy (%) | No | 350 (99.2) | 36 (100.0) | 312 (99.0) | 2 (100.0) |
|  | Yes | 3 (0.8) | 0 (0.0) | 3 (1.0) | 0 (0.0) |
| death (%) | No | 182 (97.8) | 15 (100.0) | 166 (97.6) | 1 (100.0) |
|  | Yes | 4 (2.2) | 0 (0.0) | 4 (2.4) | 0 (0.0) |
|  |  |  |  |  |  |
| p <0.05 |  |  |  |  |  |
|  |  |  |  |  |  |
| ‡ categorical variables: Fisher’s exact test. | | | |  |  |

**Supplementary Table 3. Cardiopulmonary complications among postoperative events**

| Type of complication | N (% of total complications, n=33) |
| --- | --- |
| Pneumonia | 8 (24.2) |
| Acute respiratory distress syndrome (ARDS) | 4 (12.1) |
| Respiratory failure requiring tracheostomy | 3 (9.1) |
| Death | 4 (12.1) |
| Total cardiopulmonary complication events | 19 (57.6) |

Cardiopulmonary complications were defined as pneumonia, acute respiratory distress syndrome (ARDS), respiratory failure requiring tracheostomy, and death. Percentages are calculated based on the total number of complications (n=33). Some patients experienced more than one complication; therefore, counts represent events rather than individual patients. These analyses are exploratory and should be interpreted with caution due to the limited number of events.

**Supplementary Table 4. Exploratory analysis of the association between CPET variables and cardiopulmonary complications**

| Variable | OR (95% CI) | p-value |
| --- | --- | --- |
| VO₂peak (per mL/kg/min) | 1.02 (0.94–1.10) | 0.65 |
| Anaerobic threshold (AT) | 0.35 (0.10–1.10) | 0.07 |
| VE/VCO₂ slope (per unit) | 1.06 (0.99–1.14) | 0.09 |
| AT time (per second) | 0.60 (0.13–2.70) | 0.51 |

Cardiopulmonary complications were defined as pneumonia, acute respiratory distress syndrome (ARDS), respiratory failure requiring tracheostomy, and death. Odds ratios (ORs) were estimated using exploratory logistic regression analyses. Due to the limited number of events and potential overlap of complications within individual patients, these results should be interpreted with caution.
